# The imprint of dissociative seizures on the brain

**DOI:** 10.1016/j.nicl.2024.103664

**Published:** 2024-08-29

**Authors:** S.G. Mueller, N. Garga, P. Garcia, S. Rossi, A. Vu, T. Neylan, K.D. Laxer

**Affiliations:** a Center for Imaging of Neurodegenerative Diseases, VAMC, San Francisco, CA, USA; b Dept of Radiology and Biomedical Imaging, University of California, San Francisco, CA, USA; c VA Epilepsy Center of Excellence, VAMC, San Francisco, CA, USA; d Dept. of Neurology, University of California, San Francisco, CA, USA; e VA Mental Health, VAMC San Francisco, CA, USA; f Sutter Pacific Epilepsy Program, California Pacific Medical Center, San Francisco, CA, USA

**Keywords:** Dissociative seizure, Brainstem, Resting state fMRI, Volumetry

## Abstract

**Background:**

Increased resting state functional connectivity between regions involved in emotion control with regions with other specializations, e.g. motor control (emotional hyperconnectivity) is one of the most consistent imaging findings in persons suffering from dissociative seizures (DS). The overall goal of this study was to better characterize DS-related emotional hyperconnectivity using dynamic resting state analysis combined with brainstem volumetry to investigate 1. If emotional hyperconnectivity is restricted to a single state. 2. How volume losses within the modulatory and emotional motor subnetworks of the neuromodulatory system influence the expression of the emotional hyperconnectivity.

**Methods:**

13 persons with dissociative seizures (PDS) (f/m:10/3, mean age (SD) 44.6 (11.5)) and 15 controls (CON) (f/m:10/5, mean age (SD) 41.7 (13.0)) underwent a mental health test battery and structural and functional imaging at 3 T. Deformation based morphometry was used to assess brain volume loss by extracting the mean Jacobian determinants from 457 brain, forebrain and brainstem structures. The bold signals from 445 brainstem and brain rois were extracted with CONN and a dynamic fMRI analysis combined with graph and hierarchical analysis was used to identify and characterize 9 different brain states. Welch’s t tests and Kendall tau tests were used for group comparisons and correlation analyses.

**Results:**

The duration of Brain state 6 was longer in PDS than in CON (93.1(88.3) vs. 23.4(31.2), p = 0.01) and positively correlated with higher degrees of somatization, depression, PTSD severity and dissociation. Its global connectivity was higher in PDS than CON (90.4(3.2) vs 86.5(4.2) p = 0.01) which was caused by an increased connectivity between regions involved in emotion control and regions involved in sense of agency/body control. The brainstem and brainstem-forebrain modulatory and emotional motor subnetworks of the neuromodulatory system were atrophied in PDS. Atrophy severity within the brainstem-forebrain subnetworks was correlated with state 6 dwell time (modulatory: tau = -0.295, p = 0.03; emotional motor: tau = -0.343, p = 0.015) and atrophy severity within the brainstem subnetwork with somatization severity (modulatory: tau = -0.25, p = 0.036; emotional motor: tau = -0.256, p = 0.033).

**Conclusion:**

DS-related emotional hyperconnectivity was restricted to state 6 episodes. The remaining states were not different between PDS and CON. The modulatory subnetwork synchronizes brain activity across brain regions. Atrophy and dysfunction within that subnetwork could facilitate the abnormal interaction between regions involved in emotion control with those controlling sense of agency/body ownership during state 6 and contribute to the tendency for somatization in PDS. The emotional motor subnetwork controls the activity of spinal motoneurons. Atrophy and dysfunction within this subnetwork could impair that control resulting in motor symptoms during DS. Taken together, these findings indicate that DS have a neurophysiological underpinning.

## Highlights

• The biological underpinnings of dissociative seizures (DS) are unknown.
• Dynamic task-free fMRI identified an “overshooting state (OS)” in persons with DS.
• OS shows hyperconnectivity between regions controlling emotion and sense of agency.
• Brainstem centers controlling cortical synchronization/motor function are atrophied.
• Findings indicate a mechanism for DS.

## 1 Introduction

Psychogenic non-epileptogenic seizures or dissociative seizures (DS) are defined as involuntary paroxysmal episodes characterized by disturbances of consciousness and/or motor, sensory, autonomic, cognitive or behavioral symptoms that resemble epileptic seizures but do not show the typical ictal EEG manifestations of epileptic seizures. DS are often associated with psychiatric co-morbidities, e.g., depression, anxiety or post-traumatic stress disorder (PTSD), but also with medical co-morbidities such as epilepsy, traumatic brain injury or chronic pain (Popkirov et al., 2019, Ertan et al., 2022). Their neurophysiological underpinnings are mostly unknown.

In recent years several research groups used different neuroimaging modalities to better characterize structural and functional abnormalities in DS. The findings of the studies using structural imaging were heterogeneous and occasionally even contradictory though, suggesting that DS either encompass different DS subgroups or that structural abnormalities are more representative of co-morbidities associated with DS than DS itself (Mcsweeney et al., 2017, Labate et al., 2021, Ristić et al., 2015, Sharma et al., 2022, Tsalouchidou et al., 2023). The findings from functional imaging studies were more consistent. Several resting-state fMRI studies using traditional stationary analysis methods (Amiri et al., 2021, Szaflarski et al., 2018, Allendorfer et al., 2019, Dienstag et al., 2019, van der Kruijs et al., 2012, van der Kruijs et al., 2014, Ding et al., 2013, Ding et al., 2014, Li et al., 2015a, Li et al., 2015b) for example describe what could be called emotional hyperconnectivity, i.e., an increased functional connectivity between structures involved in emotion processing/regulation that often extended to structures controlling non-emotional aspects as well. If emotional hyperconnectivity represents indeed an expression of the disturbances underlying DS, one would expect it to reflect the paroxysmal nature of DS, i.e., to vary in its expression over time. Stationary resting state fMRI analyses implicitly assume that functional interactions between brain regions are stable over the acquisition time and thus are not able to detect time-varying behavior. This is different for dynamic resting state approaches. The overall goal of this project was therefore to better characterize emotional hyperconnectivity using dynamic resting state analysis to test two main hypotheses:

1. 1 Emotional hyperconnectivity is restricted to a single state. This state represents an overshooting version of a state associated with emotion processing/control in healthy controls. Overshooting means that this state occurs more often in persons with DS (PDS) and differs from its equivalent in controls by stronger connections between regions traditionally involved in emotion processing with regions associated with other functions, e.g., motor control. The state’s expression correlates with the severity of the behavioral symptoms typically associated with DS, e.g., somatization, depression (Baroni et al., 2018, Myers et al., 2019, Duncan et al., 2018, Wang et al., 2019).
2. A disturbance, i.e., atrophy and/or hyperconnectivity**,** of the brainstem and forebrain structures that together make up the monoaminergic/cholinergic neuromodulatory system plays a major role in the expression of this overshooting state. The widespread projections of neuromodulatory system’s different subnetworks synchronize and modulate the neuronal activity across the cortical and subcortical structures (Venkatraman et al., 2017) and by extension shape the emotional and behavioral response to incoming internal and external information. In the context of DS two partially overlapping subnetworks are of particular interest. The modulatory emotion network that largely overlaps with the arousal network and influences awareness and valence of emotions and the emotional motor network that largely bypasses the voluntary motor pathways and controls the autonomic and motor aspects of emotion, e.g., smiling when being elated, and exerts an facilitatory influence on spinal motoneurons (Holstege and Kuypers, 1987, Holstege et al., 1996, Klingberg et al., 1986, Ross and Sinnamon, 1984, Ruder et al., 2021).

## 2 Methods

### 2.1 Study population

30 subjects (14 persons with DS (PDS), 16 controls (CON)) were recruited for this study. PDS were referred from 3 referral centers (VASF Medical Center, Sutter Health San Francisco, University of California, San Francisco) where they had undergone long term EEG monitoring to confirm the diagnosis of PNES without concomitant epileptic seizures. CON were recruited from the community by advertisement and had undergone a phone health screening to determine eligibility. Participants underwent imaging (see below) and a battery of structured psychological interviews: Clinician administered PTSD screen (CAPS), Dissociative Subtype of PTSD Scale (DSPS), Structured Clinical Interview for DSM-5 (SCID)) and self-report measures including (Symptom Checklist 90 (SCL90), Beck Depression Index (BDI), Pittsburgh Sleep Quality Index (PSQI), Insomnia Severity Index (ISI), Trauma History Questionnaire (THQ), and Life Experience survey (LES)). One patient was unable to undergo imaging because of claustrophobia and one control did not complete the psychological test battery. This left 13 PDS (f/m:10/3, mean age (SD) 44.6 (11.5)) and 15 CON (f/m:10/5, mean age (SD) 41.7 (13.0)).

The committee of human research at the University of California, San Francisco (UCSF) and the VA Medical Center had reviewed and approved the study. Informed consent in accordance with the Declaration from Helsinki had been obtained.

### 2.2 Imaging

#### 2.2.1 Acquisition

All 28 participants underwent imaging on a 3 T Skyra Siemens with a 32 channel head coil. The following sequences were acquired: T1 weighted image (MPRAGE, TR/TE/TI 2400/2.24/1060 msec, flip angle 8 degree, partial Fourier 1, 0.8 mm isotropic resolution, acquisition time: 6 min. T2 weighted image (T2 weighted spin echo, TR/TE 3280/564 ms, flip angle 120 degree, partial Fourier 1, 0.8 mm isotropic resolution) acquisition time: 6 min and Task-freeT2*weighted gradient echo EPI BOLD: TR 720 ms, TE 35 ms, flip angle 52°, partial Fourier 0.875, voxel size 2.5 mm isotropic, multi-band acceleration factor 6, 833 time frames. acquisition time: 10 min, 2 acquisitions.

#### 2.2.2 Image processing and analysis

##### 2.2.2.1 Structural imaging

###### 2.2.2.1.1 Brainstem

The T1 and T2 weighted image were used as input for the segmentation of 48 internal brainstem structures (Mueller, 2023). Briefly summarized, k-means clustering was used to identify 5 probabilistic brainstem and diencephalon intensity clusters corresponding to 5 brainstem tissue types from a T1, T2 and T1/T2 ratio brainstem image. SPM12′s Non-linear diffeomorphic mapping algorithm (DARTEL) was used to warp these segmentations onto a probabilistic brainstem tissue template in MNI space that has been generated from the brainstem segmentations from 100 randomly selected HCP subjects. 48 brainstem regions of interest (roi) had been manually delineated on this template using the brainstem atlases from Naidich and Duvernoy (Naidich et al., 2009) and Paxinos (Paxinos and Huang, 2011) as references: periaqueductal gray (PAG), ventral tegmental area (VTA), rostromedial tegmental (Trm) and laterodorsal tegmental (Tld) nucleus (ncl), raphe dorsalis ncl. (DR) median raphe ncl. (MedR), raphe magnus ncl. (MR), raphe obscurus (OR) and raphe pallidus ncl. (PR), left and right substantia nigra (SN), ncl. ruber (NR), ncl. pedunculopontinus (PP), ncl. reticularis cuneiformis (CR), ncl. reticularis pontis oralis (RPO), ncl. reticularis pontis tegmenti (RPT), ncl. reticularis pontis caudalis (RPC), ncl. reticularis gigantocellularis and parvocellularis (RG), ncl. reticularis medullae oblongatae (RMO), locus coeruleus (LC), ncl. subcoeruleus (SC), ncl. parabrachialis (PB), ncl. pontis (PN), ncl. tractus solitarii (NTS), ncl. olivarius inferior (OI), ventrolateral medulla (VLM), parafacial zone, (PZ) colliculus superior (CS) and colliculus inferior (CI). The transformation matrices generated during the warping step were converted into Jacobian determinant maps from which the mean intensities from each of the 48 rois were extracted for each subject. The intensities of the brainstem rois encompassing the networks of interest were averaged to obtain a single value for each network (cf. definition emotional motor brainstem network, emotional modulatory brainstem network below) for each subject.

###### 2.2.2.1.2 Cortex, subcortical structures

SPM12′s unified segmentation algorithm was used to obtain whole brain gray (gm), white (wm) and cerebrospinal fluid maps (csf). The 3 whole brain maps and the 5 brainstem segmentations were used to generate a whole brain & brainstem template with DARTEL and the Jacobian determinants calculated from the resulting transformation matrices. The mean intensities from 407 rois (366 rois from the AICHA atlas (340 cortical, 26 subcortical (hippocampus (hippo), amygdala (amy), pallidum (pall), putamen (put) combined with 32 rois from the AAL3 atlas (thalamus (thal), ncl. accumbens (nacc)), 4 rois (left and right Ch4 and Ch123) targeting the magnocellular cell groups in the human forebrain from the Juelich atlas (Joliot et al., 2015, Rolls et al., 2020, Zaborszky et al., 2008) and 6 rois that were manually drawn on the brain & brainstem template (habenula (hab), bed nucleus of stria terminalis (BNST), preoptic area (pre), lateral hypothalamus (latHT))) were extracted from the Jacobian determinant map of each subject. The intensities of the forebrain and subcortical/cortical rois encompassing the networks of interest were averaged together with the corresponding brainstem networks to obtain a single value for each network for each subject.

##### 2.2.2.2 Functional imaging

###### 2.2.2.2.1 Task-free fMRI preprocessing

Whole brain: The first 28 timeframes were discarded to allow the MRI signal to achieve T1 equilibrium. The remaining timeframes/subject underwent slice time correction, motion correction and realignment onto a mean EPI image in the T1 image subject space. Conn (Whitfield-Gabrieli and Nieto-Castanon, 2012), a SPM based toolbox was used for further processing including detection of motion outliers with its ART routine, linear detrending and band pass filtering (0.015–0.09 Hz) with simultaneous denoising. The latter includes the aCompCorr routine to reduce the effects of physiological noise (eroded white (without brainstem and diencephalon) and csf maps, 5 components each) and motion regression (6 affine motion parameters and 6 first order temporal derivatives). The transformation matrices obtained from the generation of the brain & brainstem atlas were inverted and applied to the AICHA and thalamus AAL3 rois to bring them into subject space and mean BOLD signals extracted from each frame. The roi sizes and distortions in the forebrain region did not allow to extract a reliable signal from the hab, BNST, nacc, ch4, ch123 rois and therefore were not used in the fMRI analysis.

Brainstem/diencephalon: The same brainstem/thalamus mask that was used to extract these structures from the structural images were used to extract them from the average of the denoised BOLD image (mean brainstem EPI) and each of the individual denoised timeframes. ANTS (Avants et al., 2011) was used to co-register the denoised brainstem/thalamus BOLD images onto the T1 map brainstem image in subject space. The co-registration steps used during the brainstem segmentation were inverted and concatenated to warp the rois from the brainstem template into subject space and used to extract the denoised mean BOLD signals from each frame.

###### 2.2.2.2.2 Stationary analysis

**Fig. 1:**
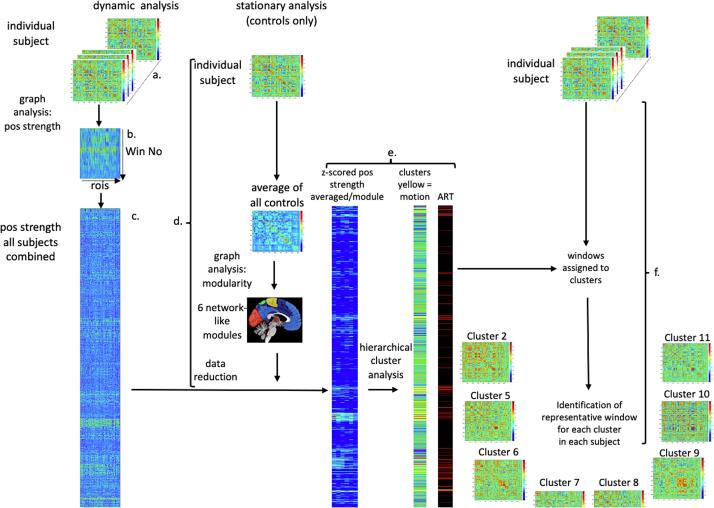
**Flow chart for dynamic fMRI processing**. A sliding windows approach was used to divide each participant’s task-free fMRI into windows for the calculation of correlation matrices (Fig. 1.a). Graph analysis was used to describe the interactions between the different rois in each window (Fig. 1b). Positive (pos) strength outputs for each window were combined to obtain a pos strength matrix for each subject followed by concatenation across subjects to obtain a population matrix that was converted into z-scores (Fig. 1c.). The roi z-scores/window were averaged across the 6 modules identified in the stationary analysis of the CON group to reduce the data in preparation for the cluster analysis, (Fig. 1d.). Hierarchical cluster analysis with each windows 6 mean z-scores as input identified 15 clusters or brain states. The ART outputs were used to identify windows with motion outliers and to calculate the % of motion outliers for each cluster. Clusters identified as motion clusters were not further evaluated (Fig. 1e). A “representative” window for each state observed in an individual was identified by calculating the Euclidian distance between each window’s correlation matrix to that of all other windows assigned to the same state in this individual and averaging these distances to obtain a representation index (RI) for each window. The window with the lowest RI was chosen as the best representation of this state in this subject and was used to characterize the networks of interest during this state (Fig. 1f.).

After scrubbing of the ART outliers, correlation matrices were calculated from the 445 de-noised BOLD time-series of each CON and averaged. The modularity_und algorithm (gamma = 1, 1000 iterations, community structure with highest Q*) (Rubinov and Sporns, 2010) from the BCT toolbox was used identify 6 modules or communities that were used in the data reduction step in the dynamic analysis (cf Fig. 1d).

###### 2.2.2.2.3 Dynamic fMRI analysis

Please see Fig. 1. Each time series was divided into sections using a sliding windows approach (window size 80 timeframes/60 sec, 729 windows/run, advanced with 1 TR) and the 445x445 correlation matrix for each window calculated (Fig. 1.a). The window size was chosen based on observations that robust estimations of the functional connectivity without loss of potentially interesting fluctuations are possible with window sizes around 30–60 s. Graph analysis was used to describe the interactions between the different rois in each window (Fig. 1b). The positive (pos) strength outputs for each window were combined to obtain a map showing the fluctuations of positive (pos) strength over the acquisition time for each roi for each subject and then concatenated across subjects to obtain population maps of pos strength for each roi (Fig. 1c.) and converted into z-scores using mean and standard deviation of the roi strength of the CON group as reference. The purpose of the z-score conversion was to standardize the data in preparation of the cluster analysis. Without that step, fluctuations in highly connected brain regions with high pos strength exert an overly strong influence on the clustering step. With that step, the fluctuations of all rois regardless of their relative connectivity contribute to the clustering. As a data reduction step in preparation for the state identification by cluster analysis, the roi z-scores/window were averaged across the 6 modules identified in the stationary analysis of the CON group (Fig. 1d.). Hierarchical cluster analysis (Ward’s minimum variance method with cubic clustering criterion to identify optimal cluster number) with each windows 6 mean z-scores as input identified 15 clusters or brain states. The ART outputs were used to identify windows with motion outliers and to calculate the % of motion outliers for each cluster (Fig. 1e). Clusters with on average more than 2 motion outliers/window were identified as motion clusters and not further evaluated. All other clusters or brain states were evaluated after excluding windows with timeframes identified as motion outliers (motion window). One person with PNES who had fewer than 1200 windows after eliminating motion clusters and motion windows had to be excluded from the analysis. Residual motion was assessed by calculating mean framewise displacement (fwd)/window and mean fwd/cluster. Eliminating windows with excessive motion results in a more rigorous elimination of motion artifacts than just eliminating the motion affected timeframe alone because it also eliminates timeframes with subthreshold motion that usually accompany timeframes with suprathreshold motion. The duration of each brain state or dwell time in a subject was calculated as the number of non-motion affected windows assigned to this state by the cluster analysis. A “representative” window for each state observed in an individual was identified by calculating the Euclidian distance between each window’s correlation matrix to that of all other windows assigned to the same state in this individual and averaging these distances to obtain a representation index (RI) for each window. The window with the lowest RI was chosen as the best representation of this state in this subject. This approach typically identifies the center window of a series of consecutive windows assigned to a state, e.g., window 34 of a series of 67 windows. Using the center slice reduces the impact of the preceding and following states on the connectivity pattern in longer series compared to shorter series which enhances the connectivity differences between longer and shorter state dwell times. The representative window was used to calculate each individual’s network connectivity by averaging each network node’s positive strength that had been derived from its correlation coefficient with each and every node within the network. Global strength was calculated by averaging of positive strength of all 445 fMRI rois.

### 2.3 Definition of networks of interest

**Fig. 2:**
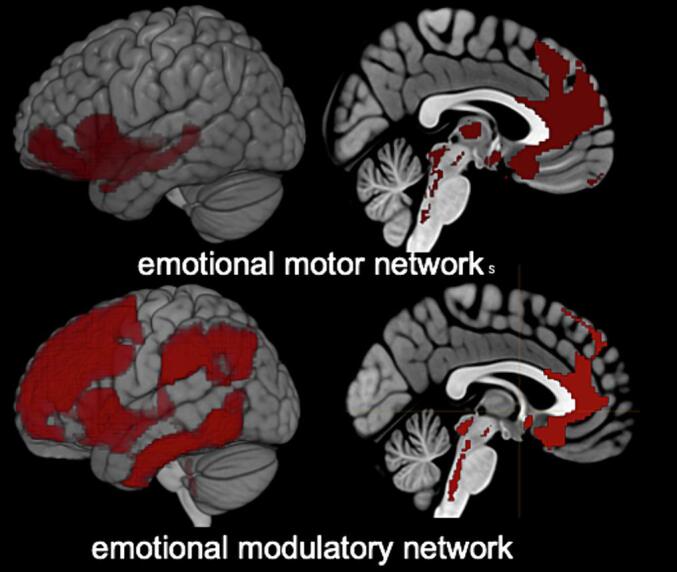
Regions defining structural and functional networks of interest. Emotional modulatory network: Brainstem: periaqueductal gray, ventral tegmental area, locus coeruleus, median raphe ncl., raphe dorsalis ncl., ncl. reticularis pontis oralis, ncl. pedunculopontinus, ncl. reticularis gigantocellularis and parvocellularis, ncl. reticularis pontis caudalis, ncl. reticularis cuneiformis, ncl. parabrachialis. Forebrain: Ch4, Ch123, ventroposterior thalamus. Cortical targets: Superior, medial, inferior frontal gyrus, supramarginal, angular gyrus, anterior insula, inferior temporal gyrus, anterior and subgenual cingulate. Emotional motor network: Brainstem: periaqueductal gray, substantia nigra, ventral tegmental area, locus coeruleus, median raphe ncl., raphe dorsalis ncl., ncl. reticularis pontis oralis, ncl. pedunculopontinus, ncl. reticularis gigantocellularis and parvocellularis, ncl. reticularis cuneiformis, ncl. parabrachialis, colliculus superior, laterodorsal tegmental ncl., ncl. subcoeruleus, rostromedial tegmental ncl.. Forebrain (fb): habenula, bed nucleus of stria terminalis, Ch123, Ch4, ncl. accumbens, preoptic area, lateral hypothalamus, medial dorsal thalamus. Cortical/subcortical targets (cortical): orbitofrontal, anterior insula, superior medial frontal, anterior cingulate, subgenual cingulate, hippocampus, amygdala.

See Fig. 2. The definition of the networks was based on the following publications: Venkatraman et al., 2017, Jhou et al., 2009, Kaufling et al., 2009, Holstege, 2009, Proulx et al., 2018, Brownstone and Chopek, 2018, Glover et al., 2023, Braine and Georges, 2023).

**Emotional modulatory network:** Brainstem: PAG, VTA, LC, MedR, DR, RPO, PP, RG, RPC, CR, PB. Forebrain: Ch4, Ch123, ventroposterior thal. Cortical targets: Superior, medial, inferior frontal gyrus, supramarginal, angular gyrus, anterior insula, inferior temporal gyrus, anterior and subgenual cingulate.

**Emotional motor network**: Brainstem: PAG, SN, VTA, LC, MedR, DR, RPO, PP, RG, CR, PB, CS, Tld, SC, Trm. Forebrain (fb): hab, BNST, Ch123, Ch4, nacc, pre, latH, medial dorsal thal. Cortical/subcortical targets (cortical): orbitofrontal, anterior insula, superior medial frontal, anterior cingulate, subgenual cingulate, hippo, amy.

### 2.4 Statistics

Welch t-tests were used to investigate the differences between PDS and CON as outlined in the introduction. Two-tailed tests were used because even though there was an a priori hypothesis regrading the behavior of the overshooting state, i.e., longer dwell time and more pronounced network hyperconnectivity in PDS than CON, this was not the case for the other states. They were expected to show no group differences, but the direction of potential group differences was unknown and thus two-tailed tests were deemed most appropriate to demonstrate the unique behavior of the overshooting state. Given the a priori hypotheses regarding the directions of behavior with imaging associations (negative for atrophy – behavioral associations and positive for hyperconnectivity – behavioral associations) one-tailed Kendall tau correlations were used to investigate those. False Discovery Rate (FDR) (q = 0.05) was used to correct for multiple comparisons for all tests for which no a priori hypotheses existed (cf. Introduction) existed.

## 3 Results

### 3.1 Population characteristics

Table 1. summarizes demographic and behavioral characteristics. PDS had worse mental and general health scores than CON. The measures for somatization, depression and anxiety from the SCL90, DSPS severity and CAPS current were chosen from the test battery as measures of interest because they captured co-morbidities thought to be contributing to DS and in the case of the SCL90 were obtained within less than 24 h of the MRI exam, i.e., representative for the participants emotional state during the MRI. All but one PDS suffered from more than one type of seizures of which at least one was associated with motor manifestations. Most PDS did either not keep a seizure diary or did not differentiate by seizure type which prevented a meaningful investigation of the relationship between imaging features and seizure frequency.

**Table 1:** Study Population: Characteristics.

		**CON**		**PDS**		**p (Welch test)**
		n = 15		n = 13		
Gender (f/m)		10/5		10/3		
Age		41.7 (13.0)		44.6 (11.5)		0.564
BDI		3.07 (4.64)		14.8 (8.23)		<0.001
SOMATIZATION SCL90		1.47 (2.29)		17.6 (6.68)		<0.001
OBSESSIVE-COMPULSIVE SCL90		3.80 (3.84)		16.2 (8.83)		<0.001
INTERPERSONAL SENSIBILITY SCL90		2.53 (3.98)		6.62 (5.06)		0.02
DEPRESSION SCL90		4.73 (7.69)		17.5 (11.4)		0.001
ANXIETY SCL90		1.67 (2.79)		11.8 (6.61)		<0.001
ANGER-HOSTILITY SCL90		0.733 (1.2)		2.31 (1.93)		0.01
PHOBIC-ANXIETY SCL90		0.667 (1.40)		6.85 (6.43)		0.002
PARANOID-IDEATION SCL90		0.933 (1.49)		3.92 (3.23)		0.008
PSYCHOTICISM SCL90		0.933 (1.53)		6.46 (4.01)		<0.001
Total SCL90		20.4 (27.2)		101 (43.8)		<0.001
DSPS lifetime		0.600 (1.40)		4.77 (4.09)		<0.001
DSPS severity		0.133 (0.516)		4.77 (4.09)		<0.001
CAPS curr Total		0.600 (1.59)		25.1 (17.8)		<0.001
CAPS life Total		2.47 (4.10)		36.1 (21.2)		<0.001
STAI Y1		30.9 (11.1)		41.0 (10.9)		0.021
STAI Y2		34.6 (10.0)		46.6 (11.8)		0.013
Total TQH		24.1 (40.5)		71.7 (81.8)		0.009
LES Total		−3.73 (9.67)		−0.308 (6.98)		0.474
Health Survey PCS-12		54.4 (4.97)		32.1 (9.82)		<0.001
Health Survey MCS-12		51.1 (10.5)		40.4 (13.8)		0.012
PSQI		4.80 (1.61)		11.6 (2.81)		<0.001
PSQI-A Total		1.60 (2.20)		6.62 (4.13)		0.001
ISI Total		4.67 (4.34)		15.6 (6.14)		<0.001

BDI, Beck Depression Index; SCL90, Symptom Checklist 90; DSPS, Dissociative Subtype of PTSD; CAPS, Clinician administered PTSD screen; STAI, State Trait Anxiety Inventory; TQH, Trauma History Questionnaire; LES, Life Experience Survey; PSQI, Pittsburgh Sleep Index; ISI, Insomnia Severity Index, CON, controls; PDS, persons with dissociative seizures.

### 3.2 Structural imaging

None of the cortical, subcortical or brainstem roi (n = 456) volume differences between the two groups survived correction for multiple comparisons. The emotional modulatory brainstem network (0.69 (0.05) vs. 0.73 (0.02) p = 0.02) and emotional modulatory brainstem-forebrain (0.74 (0.05) vs. 0.77 (0.04) p = 0.01) network but not the emotional brainstem-forebrain-cortical network (0.92 (0.06) vs. 0.93 (0.05) p = 0.27) were smaller in PDS than in CON. The same applied to the emotional motor brainstem network (0.71 (0.04) vs. 0.74 (0.05), p = 0.02) and the emotional motor brainstem-forebrain network (0.77 (0.04) vs. 0.80 (0.02), p = 0.02) in PDS compared to CON. The emotional motor brainstem-forebrain-cortical network (0.87 (0.04) vs. 0.89 (0.04), p = 0.21) was not different between the two groups.

Table 2 summarizes the associations between network volume reductions and behavioral measures. Volume loss within the brainstem emotional modulatory network correlated with somatization and brainstem-forebrain modulatory emotional network with somatization and depression severity. Volume loss within the emotional motor brainstem network correlated with somatization severity and volume loss within the emotional motor brainstem-forebrain network with dissociation severity.

**Table 2:** Associations network between volume reductions and behavioral measures of emotional distress.

				bs emotional modulatory	bs-fb emotional modulatory	bs-fb-cortex emotional modulatory	bs emotional motor	bs-fb emotional motor	bs-fb-cortex emotional motor
SOMATIZATION SCL90		Kendall's Tau B		−0.289	−0.25	0.042	−0.256	−0.222	−0.048
		p-value		0.019**	0.036*	0.619	0.033**	0.056	0.366
DEPRESSION SCL90		Kendall's Tau B		−0.189	−0.233	0.074	−0.195	−0.189	0.047
		p-value		0.084	0.045*	0.705	0.078	0.084	0.633
CAPS curr Total		Kendall's Tau B		−0.094	−0.117	0.012	−0.088	−0.158	−0.059
		p-value		0.254	0.204	0.533	0.268	0.132	0.34
DSPS severity		Kendall's Tau B		−0.169	−0.189	0.129	−0.129	−0.262	0.036
		p-value		0.125	0.1	0.81	0.19	0.038**	0.598
									

** p < 0.05, one-tailed with FDR correction, *, p < 0.05 without FDR correction

bs, brainstem; fb, forebrain; cortex, cortical target regions.

### 3.3 Resting state fMRI dynamic analysis

**Fig. 3:**
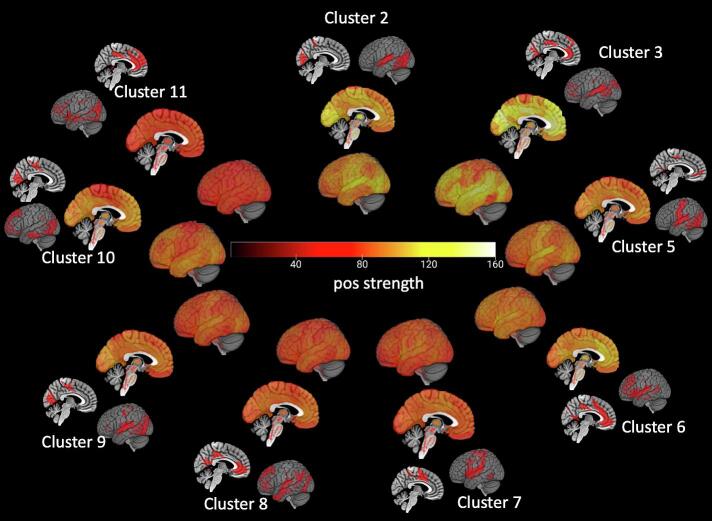
**Brain states identified by dynamic task-free fMRI analysis**. Each state is described by its connectivity strength inner circles (positive strength in whole brain analysis, yellow colors indicating regions with higher connectivity) and state specific hub regions (outer regions), i.e., rois whose positive exceeds the 90th percentile, displayed in red. (For interpretation of the references to colour in this figure legend, the reader is referred to the web version of this article.)

Cluster analysis detected 15 different clusters or states of which 6 fulfilled the criteria for motion clusters and were excluded. The remaining states were characterized by calculating the mean positive strength for each roi across the whole population and state specific hub regions., i.e., high connectivity rois whose mean positive strength exceeded the 90th percentile (see Fig. 3).

**Fig. 4:**
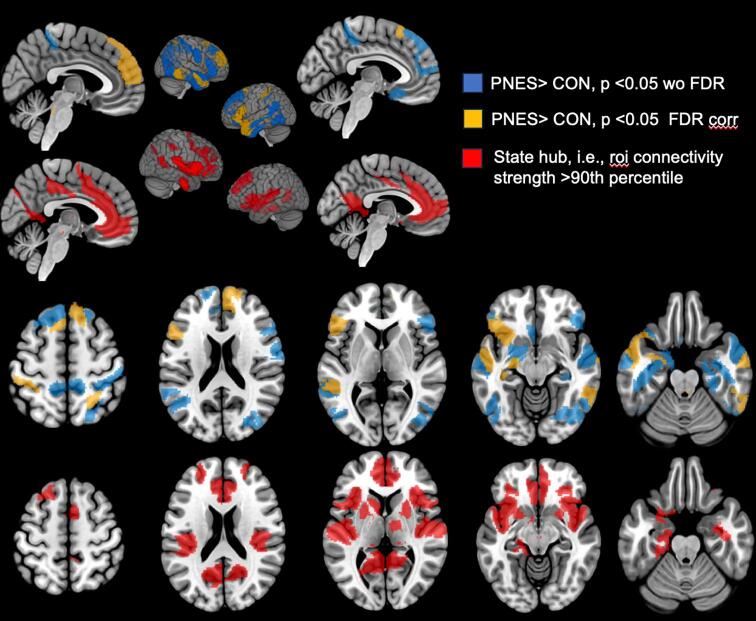
**Overshooting state (state 6) details.** First and third rows show rois with higher connectivity (pos strength) in PDS than CON with (yellow) and without (blue) correction for multiple comparison. Second and fourth rows show corresponding state 6 hub rois. With few exceptions (rois in anterior insula, inferior temporal lobe, medial frontal superior gyrus) there is no overlap between state hub regions and sig. pos strength difference regions. (For interpretation of the references to colour in this figure legend, the reader is referred to the web version of this article.)

Please see Fig. 4. and Table 3. State 6 was the only state that fulfilled all criteria of the hypothesized overshooting state. It had a longer dwell time (p = 0.01) in PDS, an increased mean global connectivity (p = 0.01), and an increased within connectivity in the bs-cortical emotional motor network (p = 0.01) and the bs-cortical emotional modulatory network (p = 0.04) in PDS compared to CON. Rois with increased connectivity compared to CON (p < 0.05, q = 0.05 FDR corrected) encompassed rois located in supplementary motor region, medial frontal superior, frontal opercular, anterior insula, middle and inferior temporal, and postcentral and superior parietal cortices that were often accompanied by less prominent hyperconnectivity (p < 0.05 without FDR correction) in opposite frontal, temporal and parietal regions but mostly spared the state 6 state hubs that included subgenual, anterior and mid cingulate, dorsolateral frontal and insular cortices, i.e., structures known to be involved in emotion control, and also subcortical structures such as caudate, putamen and thalamus (see Fig. 3). Limiting the connectivity analysis to the connectivity between rois with significant group differences in the whole brain connectivity analysis (blue and yellow in Fig. 3) and state 6 hub regions (red in Fig. 3) showed an increased connectivity between these rois in PDS compared to CON (1.70 (0.28) vs 1.23 (0.19) p = 0.0004).

**Table 3:** Characteristics of resting state fMRI states: Dwell Time and positive Strength.

	Group	No subs with state	Dwell time	Connectivity:	global	bs emotional modulatory	bs-cortex emotional modulatory	bs emotional motor	bs-cortex emotional motor
State 2	CON	6	37.8 (76.7)		95.14 (2.73)	0.13 (0.03)	4.33 (0.22)	0.28 (0.03)	2.33 (0.21)
	PDS	9	56.3 (54.6)		96.66 (2.95)	0.12 (0.02)	4.87 (0.29)*	0.29 (0.04)	2.36 (0.36)
State 3	CON	5	24.3 (53.1)		100.44 (7.70)	0.12 (0.03)	4.86 (0.81)	0.29 (0.06)	2.46 (0.26)
	PDS	2	5.2 (14.1)		97.44 (2.93)	0.11 (0.01)	5.51 (0.12)	0.26 (0.02)	2.40 (0.08)
State 5	CON	14	152.9 (132.1)		86.03 (2.90)	0.12 (0.02)	4.30 (0.30)	0.27 (0.04)	2.16 (0.18)
	PDS	12	134.8 (65.3)		87.27 (4.00)	0.13 (0.03)	4.52 (0.52)	0.29 (0.05)	2.22 (0.21)
**State 6**	CON	11	23.4 (31.2)		**86.53 (4.15)**	0.13 (0.03)	**4.74 (0.60)**	0.30 (0.05)	**2.45 (0.23)**
	PDS	**10**	93.1 (88.3)*		90.38 (3.21)*	0.14 (0.04)	5.24 (0.07)*	0.31 (0.67)	2.71 (0.22)*
State 7	CON	14	148.9 (94.0)		77.10 (1.87)	0.12 (0.02)	4.17 (0.41)	0.28 (0.05)	2.06 (0.18)
	PDS	12	213.3 (113.4)		76.24 (2.36)	0.11 (0.01)	4.14 (0.43)	0.26 (0.03)	2.09 (0.17)
State 8	CON	15	245.5 (153.0)		75.15 (2.28)	0.12 (0.01)	3.90 (0.29)	0.27 (0.04)	2.16 (0.15)
	PDS	10	146.6 (146.2)		76.46 (3.10)	0.11 (0.01)	4.10 (0.34)	0.25 (0.03)	2.07 (0.33)
State 9	CON	14	151.9 (94.4)		79.28 (2.32)	0.13 (0.02)	3.97 (0.38)	0.28 (0.04)	2.11 (0.26)
	PDS	11	105.3 (87.6)		80.18 (2.89)	0.13 (0.04)	4.31 (0.43)*	0.30 (0.09)	2.18 (0.27)
State 10	CON	7	33.7 (52.7)		82.84 (5.89)	0.11 (0.02)	4.26 (0.23)	0.26 (0.03)	2.24 (0.20)
	PDS	9	72.5 (83.4)		83.26 (3.55)	0.13 (0.04)	4.65 (0.54)	0.30 (0.08)	2.39 (0.33)
State 11	CON	15	364.0 (238.0)		69.89 (2.34)	0.12 (0.02)	3.93 (0.36)	0.27 (0.03)	2.05 (0.16)
	PDS	12	243.1 (167.4)		70.46 (2.38)	0.10 (0.01)	3.88 (0.30)	0.25 (0.02)	1.97 (0.18)

* p < 0.05, two-tailed.

bs, brainstem; fb, forebrain; cortex, cortical target regions; CON, controls; PDS, persons with dissociative seizures.

State 6 dwell time was negatively correlated with volume loss within the emotional modulatory brainstem-forebrain (Kendall tau = -0.295, p = **0.032**) and emotional motor brainstem-forebrain network (Kendall tau = -0.343, p = 0.015). Finally, state 6 dwell time was also positively correlated with somatization (Kendall tau = 0.343, p = 0.017), depression (Kendall tau = 0.434, p = 0.004), and CAPS current (Kendall tau = 0.36, p = 0.015) but not DSPS severity (Kendall tau = 0.276, p = 0.053). None of the other states showed these associations.

## 4 Discussion

The study had two major findings. 1. Dynamic task-free fMRI analysis identified a brain state (state 6) whose features met all the core characteristics of the hypothesized overshooting state in PDS, i.e., more common and hyperconnected in PDS, regions involved in emotional control identical with state hub rois, dwell time associated with behavioral measures of emotional stress. None of the other states met these criteria. 2. PDS was characterized by volume loss in the modulatory and emotional motor brainstem subnetworks and the modulatory and emotional motor brainstem-forebrain subnetworks of the neuromodulatory system but spared their cortical and subcortical target areas. The severity of these volume losses correlated with behavioral measures of emotional stress and expression of the overshooting state.

In sum, the findings of this project indicate that DS have a biological underpinning. Potential implications regarding DS mechanism and treatment will be discussed in more detail in the next paragraphs.

The first major finding is the identification of a task-free brain state that met the characteristics of the hypothesized overshooting state. This was not unexpected. Several previous studies that used a variety of stationary analysis approaches described an emotional hyperconnectivity characterized by an increased functional connectivity between regions involved in emotional control but also with regions with other specializations, e.g., motor control (Amiri et al., 2021, Szaflarski et al., 2018, Allendorfer et al., 2019, Dienstag et al., 2019, van der Kruijs et al., 2012, van der Kruijs et al., 2014, Ding et al., 2013, Ding et al., 2014, Li et al., 2015a, Li et al., 2015b). Given that its dwell time was longer than that of other two hyperconnectivity states (state 2 and 3) and that it was the only state that not only had an increased global strength but also a longer dwell time in PDS, it seems plausible to assume that the overshooting state represents the dynamic equivalent of the stationary emotional hyperconnectivity. The new insight gained by the dynamic analysis is that functional brain connectivity in PDS does not differ from that in CON with exception of the overshooting state. This also implies that isolating these episodes from normal “background” task-free activity states by a dynamic analysis allows for a better characterization of the overshooting state’s nature. As predicted, the overshooting state’s state hubs, i.e., rois whose connectivity were within the top 10 percent, corresponded to regions known to be involved in emotional processing. Interestingly, except for three small rois, the connectivity between state hub rois was not different between the two groups. However, the connectivity of state hub rois with hyperconnected non-hub rois in PDS was increased indicating a stronger interaction between them in PDS. In addition to regions involved in motor control, e.g., supplementary motor cortex, precentral gyrus, non-hub rois with increased connectivity in PDS included the postcentral gyrus, anterior insula, medial frontal superior cortex, angular gyrus, superior and middle temporal gyrus, i.e., brain regions that have been linked to body ownership and/or sense of agency (Harduf et al., 2023, Haggard, 2017). Given the association between overshooting state dwell time and the severity of psychiatric symptoms, it is tempting to speculate that stressful events affect the interaction between emotional hub rois and non-hub rois controlling sense of agency and body ownership. This will likely not only increase overshooting state severity and dwell time but also result in an impaired sense of body ownership or sense of agency that could explain the increased interictal somatization and dissociation reported by PDS. It is even possible that the overshooting state could cause the DS if it is assumed that there are limits to the time it can be sustained and/or the degree of global hyperconnectivity it can reach. The DS would then be triggered when these limits are reached or surpassed, and its semiology be shaped by the configuration of the individual’s overshooting state at that time. The DS episode would either resolve the overshooting state completely or at least lower its intensity below the critical threshold. This would bring a temporary relief of the interictal symptoms (Hopp et al., 2022) that lasts until new or ongoing stressful experiences re-initiate or re-enforce the overshooting state again. This would mean that DS represent a release mechanism that is triggered when the stress-induced overshooting state can no longer be sustained.

The second major finding was the demonstration of volume losses within the monoaminergic and cholinergic neuromodulatory system in PDS. The etiology of these volume losses is not known. However, considering that chronic stress, adverse life experiences and also mild TBI are known to cause atrophy in several brain regions including the brainstem (Morin et al., 2020, Howell et al., 2013, Knutsen et al., 2020, Kim et al., 2021, Kulkarni et al., 2019, McPherson et al., 2024., Kawa et al., 2020), it seems likely that atrophic processes are responsible for the volume losses although pre-existing factors, i.e., genetic factors, could play a role as well (Leu et al., 2020, Jungilligens et al., 2022). The atrophy was most pronounced within the brainstem and brainstem-forebrain components of the modulatory emotion and the emotional motor networks and was accompanied by an increased functional connectivity between these subnetworks and their cortical and subcortical target regions during the overshooting state. Intriguingly, these structural findings suggest a potential mechanism for DS. The modulatory emotion subnetwork controls awareness and valence of experiences by synchronizing the activity across cortical and subcortical regions (van den Brink et al., 2019). Atrophy within crucial elements of this subnetwork, e.g., PAG or LC, could therefore facilitate the overshooting by initiating and maintaining the enhanced interictal interaction between state hub rois and non-hub rois. The subnetwork’s increased functional connectivity during the overshooting state and the association between subnetwork atrophy with overshooting dwell time support this hypothesis. The emotional motor subnetwork exerts a facilitatory influence on motoneurons in the spine. Atrophy within this subnetwork, particularly within its motor nuclei PP and CR, could impact that control which then together with the subnetwork hyperconnectivity during the overshooting state could explain motor symptoms during DS episodes.

The atrophy within these two subnetworks could also explain the prevalence of certain co-morbidities and the female preponderance of DS. Most of the brainstem and forebrain structures belonging to the emotional motor and modulatory networks participate also in other functions. DR and MR for example are core structures of networks controlling mood and PAG is an important component of subnetworks involved in fear and pain processing. This “multitasking” ability of DS affected brainstem and forebrain structures could be the reason for the high prevalence of depression, anxiety disorders or chronic pain in PDS. Finally, sex-specific differences of the neuromodulatory system at the molecular and anatomical level are well documented in animal models (Kawa et al., 2020, Yu et al., 2021, Philippe et al., 2022, Liiver et al., 2023, Kim et al., 2023, Bates et al., 2023 Feb. Sun et al., 2020) and are likely to exist in humans as well. It is tempting to speculate that such differences could explain the predominance of DS in women.

The findings of this study could also have implications for the treatment of DS. Treating co-morbidities such as PTSD or depression or improving coping with stressful events by various psychotherapeutic approaches and/or antidepressant medication are currently the mainstay in DS treatment (Cobb et al., 2023, Chen and LaFrance, 2016, Young et al., 2023). Despite targeting and often improving some of the DS core symptoms, the success of these approaches regarding DS control is only moderate (Cobb et al., 2023). If the overshooting state described in this study plays indeed the role outlined in the previous paragraphs, one must assume that the current treatment approaches also have no or only a moderate effect on its expression. It also raises the question if the presence of the overshooting state reduces the efficacy of psychotherapy and drug treatment. If this is true, it would mean that it is first necessary to “reset” the overshooting state for the drug and psychotherapy to become effective. Transcranial magnetic stimulation (TMS) has been shown to reduce hyperconnectivity in stationary task-free fMRI and to improve symptoms in major depression, anxiety and other functional neurological disorders (Kim et al., 2023a, Ge et al., 2020). This suggests that it could also have beneficial effects on the overshooting state and by extension seizure control in PDS without concomitant epilepsy. Two small pilot studies that showed an improved seizure control after repetitive TMS in PDS support that notion (Parain and Chastan, 2014, Peterson et al., 2018). Mapping an individual patient’s overshooting state with dynamic task-free fMRI could help to identify the optimal stimulation target and thus maximize the treatment effect.

The study has several limitations. 1. The most severe limitation is the small sample size. The inclusion criteria for patients required the demonstration of DS without epileptic seizures by video-EEG monitoring and prohibited the inclusion of patients with a history of moderate-severe TBI, history of a psychiatric or neurological disease except for known DS comorbidities or an abnormal MRI which limited the number of qualifying patients. The COVID related restrictions reduced that number further. The findings of this study need to be confirmed in a larger patient population that should also include patients with DS and epilepsy. 2. The fMRI parameters were optimized for dynamic imaging with TRs shorter than 1 sec. This resulted in a larger voxel size than what would have been optimal for the small brainstem structures. It is possible that this influenced the fMRI findings in the brainstem.

## Funding

This work was supported by an award from the US Department of Defense (W81XWH-17–1-0336) to SGM.

## CRediT authorship contribution statement

**S.G. Mueller:** Writing – review & editing, Writing – original draft, Methodology, Investigation, Funding acquisition, Formal analysis, Conceptualization. **N. Garga:** Writing – review & editing, Conceptualization. **P. Garcia:** Writing – review & editing. **S. Rossi:** Writing – review & editing, Data curation. **A. Vu:** Writing – review & editing. **T. Neylan:** Writing – review & editing, Conceptualization. **K.D. Laxer:** Writing – review & editing, Conceptualization.

## Declaration of competing interest

The authors declare that they have no known competing financial interests or personal relationships that could have appeared to influence the work reported in this paper.

## Data Availability

Data will be made available on request.
